# Potential Benefits of Flavonoids on the Progression of Atherosclerosis by Their Effect on Vascular Smooth Muscle Excitability

**DOI:** 10.3390/molecules26123557

**Published:** 2021-06-10

**Authors:** Rosa Edith Grijalva-Guiza, Aura Matilde Jiménez-Garduño, Luis Ricardo Hernández

**Affiliations:** 1 Departamento de Ciencias Químico Biológicas, Universidad de las Américas Puebla, San Andrés Cholula 72810, Mexico; rosa.grijalvaga@udlap.mx; 2 Departamento de Ciencias de la Salud, Universidad de las Américas Puebla, San Andrés Cholula 72810, Mexico; aura.jimenez@udlap.mx

**Keywords:** flavonoids, progression, atherosclerosis, ion channels, vascular, smooth muscle, calcium currents, oxidative stress, membrane potential, excitability

## Abstract

Flavonoids are a group of secondary metabolites derived from plant-based foods, and they offer many health benefits in different stages of several diseases. This review will focus on their effects on ion channels expressed in vascular smooth muscle during atherosclerosis. Since ion channels can be regulated by redox potential, it is expected that during the onset of oxidative stress-related diseases, ion channels present changes in their conductive activity, impacting the progression of the disease. A typical oxidative stress-related condition is atherosclerosis, which involves the dysfunction of vascular smooth muscle. We aim to present the state of the art on how redox potential affects vascular smooth muscle ion channel function and summarize if the benefits observed in this disease by using flavonoids involve restoring the ion channel activity.

## 1. Introduction

Atherosclerosis is the principal cause of cardiovascular diseases [1,2]; these diseases are considered the first cause of mortality globally, especially in developed and high-income countries, taking the lives of almost 18 million people every year [3]. Atherosclerosis is a disease that progresses over the years and can affect anybody, without race or gender restriction; however, genetic and environmental factors place specific populations at risk [4]. The complications of this disease also represent high expenses for patients and healthcare systems. Therefore, it is urgent to make efforts to prevent the progression of this disease [3,5,6]. The primary preventive measures include the improvement of nutritional habits. Diets enriched in polyunsaturated fatty acids and antioxidants with proper fiber intake and low in processed foods and saturated fatty acids have been associated with a lower incidence of cardiovascular diseases. One example is the consumption of antioxidants, such as ascorbic acid, carotenoids, vitamin E, and polyphenols. Many studies indicate the benefits of consuming fruits and vegetables containing antioxidants known as flavonoids [7,8].

Flavonoids are secondary metabolites from plants and the biggest group of polyphenolic compounds. There are more than 5000 different flavonoids with diverse activities. Polyphenols have been studied a lot, ever since Albert Szent-Györgyi discovered them in 1930 when he isolated citrine from lemon and called it vitamin P. This name was chosen because the molecule regulated the permeability of capillaries [9]. We consume flavonoids in our regular diet when we eat fruits and vegetables every day [10]. They are subdivided into subgroups: chalcones, aurones, flavones, flavanols, anthocyanidins, flavonols, flavanones, and isoflavones [11]. They have common characteristics that give them a high nutritional value, although they present differences in absorption, metabolism, and bioavailability [12]; they all have substantial benefits for human health if consumed regularly. The help in vascular health results from their biological activities as antioxidants since the opposition to oxidative stress lowers the risk of atherosclerosis development [13]. Some of these actions are derived from their interaction with reactive oxygen species (ROS) and reactive nitrogen species (RNS); however, effects on ion channels in the plasmatic membrane of endothelial and vascular smooth muscle cells have also been reported [13,14].

Ion channels are integral proteins in plasmatic and inner membranes. They are responsible for the ion movement across membranes called ion currents. These currents produce changes in the electrical properties of cells such as endothelial and vascular smooth muscle cells (VSMCs) in vessels. Many of these imbalances affect intracellular calcium concentrations, disturbing the vessels’ contraction–relaxation regulation [14]. VSMCs constitute wall vessels and control the diameter of medium and large blood arteries. This group of cells contracts or relaxes to keep blood pressure and oxygenation of tissues. When vascular vessels start accumulating oxidized cholesterol, atherosclerosis develops, causing cardiac complications and different peripheral vascular diseases with high morbidity and mortality rates [11,15].

This paper discusses the influence of vascular smooth muscle ion currents in the progression of atherosclerosis and how this altered condition can be reversed using flavonoids.

## 2. Atherosclerosis

Atherosclerosis is an immune-metabolic disease because it involves cells of the immune system and organic molecules of metabolism. Atherosclerotic lesions show high amounts of monocytes, macrophages, lipoproteins, and low-density cholesterol. The progress of the disease is considered chronic. It involves a degenerative process that occurs in many phases. Damage is generated in the walls of blood vessels due to the accumulation of lipids, calcium, platelets, and other blood compounds [16]. The process of plaque development takes place in the coronary, aorta, carotid, iliac, and femoral arteries for many years [17]. The process initiates with an early fatty streak development during childhood; then, an early fibroatheroma is formed during adolescence and the twenties. Advanced atheroma or a thin cap of fibroatheroma occurs in elders above 55 years [18].

### 2.1. General Concepts

**Figure 1:**
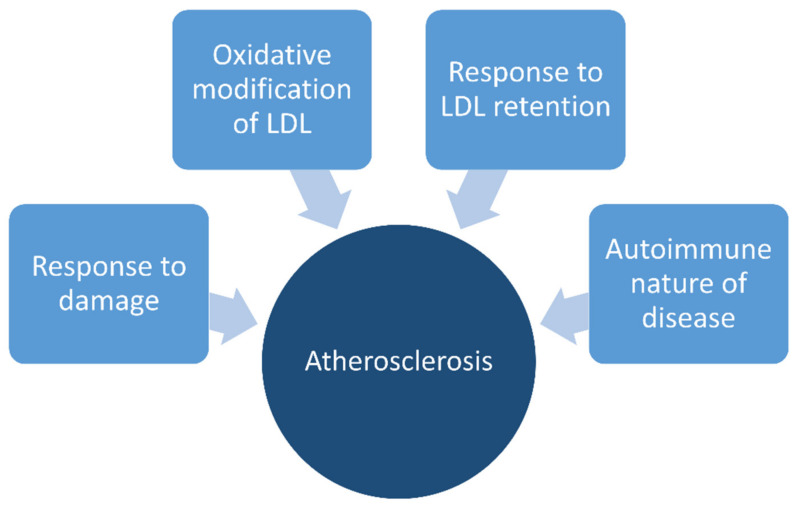
Hypotheses of the pathogenesis of atherosclerosis.

The pathogenesis of atherosclerosis can be resumed in four hypotheses: (a) oxidative modification of low-density lipoproteins (LDL) [19,20], (b) response to damage [21], (c) response to LDL retention [22], and (d) autoimmune nature of the disease [23,24] (Figure 1). Two experiments support the oxidative LDL modification hypothesis: firstly, it was demonstrated that oxidized-LDL (ox-LDL) causes damage to cultured endothelial cells [25,26]; secondly, ox-LDL was recognized by different scavenger receptors (Lectin-like oxidized LDL receptor-1 (LOX-1) including scavenger receptors that bind LDL (CD36), scavenger receptors for phosphatidylserine and oxidized LDL in human atherosclerotic lesions (SR-PSOX), and the multifunctional receptor in atherosclerosis (SR-A macrophage receptors)), which mediates the influx of lipids into macrophages. Activation of all these receptors promoted the formation of foam cells in cultured endothelial cells [27]. 

The second hypothesis considers that the damage caused to the vascular endothelium is responsible for the endothelial activation and the initiation of the atherosclerotic process. The process included an increased permeability of lipoproteins and the expression of adhesion molecules, such as E-selectin, P-selectin, vascular endothelial cell adhesion molecule-1 (VCAM-1), and intercellular adhesion molecule-1 (ICAM-1). These molecules bind to their corresponding receptors on circulating monocytes and T lymphocytes and induce the recruitment of these cells to the site of injury, where ROS species from fibroblast and other cells cause atherogenesis [28].

The third hypothesis considers the retention of LDL firstly. The accumulation of lipoproteins within the arterial wall and arterial proteoglycans can trigger the pro-inflammatory cascade and promote atherosclerosis [29,30]. The fourth hypothesis is related to the disease’s autoimmune nature; this involves the immune response before plaque development. During the onset of the illness, antigens, antibody complexes, T lymphocytes, B lymphocytes, and proteins of the complement system participate, and infiltration of mononuclear cells into the lesion, such as CD8^+^ lymphocytes, CD4^+^ (Th1) helper T lymphocytes, mast cells, monocytes, and macrophages, occurs. DAMPs (damage-associated molecule patterns) such as heat stress proteins (HSP) and ox-LDL appear; in fact, anti-HSP60 antibodies can be used as a disease marker for progression. Innate immune cells recognize ox-LDL and HSPs and activate inflammation; all these events support the importance of immunity during disease development [31,32,33].

Any of these four processes cause atherosclerosis, but it can be prevented by diminishing risk factors. The major atherogenic risk factors are central obesity, oxidative stress, dyslipidemia, hyperglycemia, and pro-inflammatory states [34,35]. Studies have reported that high serum concentrations of LDL-cholesterol, glucose, and C-reactive protein (CRP) are directly associated with the risk of developing cardiovascular disease [35,36].

It is necessary to understand the details of the disease’s pathophysiology to develop appropriate preventive and/or therapeutic strategies that can avoid vascular calcification [37]. During this process, the predominant cell type in the arterial wall is smooth muscle cells; they are responsible for vessels’ structure and function integrity [21]. Calcification is generated in the intima of blood vessels at specific points that form crystal patches with necrotic core spaces [37,38]. During the initial atherosclerotic stage, smooth muscle cells are 90% of the cellular content in the lesion area. However, this changes in advanced lesions; in those cases, the extracellular matrix predominates over smooth muscle cells, forming the fibrous covering of plaques. Smooth muscle cells with a non-proliferative contractile phenotype are transformed into cells that actively proliferate, migrate attracted by chemotactic agents, and produce extracellular matrix proteins (collagen, elastin, and proteoglycans). This transformation activates the expression of genes that encode membrane receptors for growth factors [22]. The migration of smooth muscle cells promotes calcification in the injured area, which is associated with higher mortality and morbidity rates [39].

The spontaneous rupture of an atherosclerotic plaque causes the activation of prothrombotic elements of the endothelium. When platelets aggregate, they release their granules rich in mitogens and induce the migration and proliferation of smooth muscle cells, including inflammation and oxidative stress, which are present during all stages of the disease [40,41].

### 2.2. Stages of Atherosclerosis

There is a way to classify the progression of atherosclerosis based on histological studies from human and animal autopsies in the following stages: pre-atherosclerosis, early atherosclerosis, late atherosclerosis, and clinical sequelae [42,43]. In all phases, vascular smooth muscle cells are crucial for plaque development. Pre-atherosclerosis initiates at birth because the diffuse intimal thickenings and intimal xanthomas are used as an adaptation to the blood flow [44,45,46]; this is considered a pre-plaque [42]. During early atherosclerosis, pathological intima thickening is formed. This early plaque contains extracellular lipid pools deep in the intima with a large quantity of VSMCs and extracellular matrix (ECM) [42,43]. The progression involves the retention and oxidation of LDL, induction of inflammation, and VSMCs proliferation, with phenotypic changes and death [22,47]. VSMCs produce ECM in the intima, where it plays an essential role in the initiation of atherosclerosis. During this process, it has been demonstrated that negatively charged side chains of proteoglycans interact with the positively charged side of apolipoproteins [48] to retain lipoproteins from plasma [30]. The trapped lipoproteins suffer oxidation, macrophages are recruited, and inflammation initiates [22]. Sometimes, a micro-calcification near media tissue occurs, which has been associated with VSMC apoptosis [49]. Pathological intima thickening in late stages always presents with abundant macrophages, representing a crucial step for the progression to fibroatheroma [50,51,52,53,54] and VSMCs’ proliferation, migration, and phenotype change [55]. During the late stages, the accumulation of macrophages in the luminal space is necessary. The lesion is characterized by a fibrous cap and necrotic core, which is formed by dead VSMCs and macrophages that phagocyte lipids and become foam cells [56,57]; then, the fibroatheroma develops, and calcification can be observed first in the necrotic core and then in the surrounding ECM [58,59,60]. This mature plaque forms sheets whose fragments can protrude into the lumen and precipitate thrombosis [42,60]. Finally, clinical sequelae depend on which artery has been affected [61].

### 2.3. Role of Oxidation

ROS and RNS species are produced in low concentrations in VSMCs, adventitia, and endothelial cells in normal conditions. They function as mediators in cell signaling to regulate vascular activity [13,62,63,64], participate in vascular smooth muscle growth, and regulate contraction and relaxation [65,66]. However, during pathological states, there is a disequilibrium between antioxidants and oxidants, and when oxidants are favored, oxidative stress is produced. The sources of ROS include lipooxygenases, cytochrome P450, cyclooxygenase, xanthine oxidase, mitochondrial respiration, NADPH oxidase, and uncoupled nitric oxide synthases [13]. Additionally, intracellular ROS production can derive from the electron-transport chain [67]. One of the first tissues affected in atherosclerosis is the endothelium, where nitric oxide (NO), endothelin I, angiotensin II, adhesion molecules, and cytokines are produced [13,68]. Oxidative stress affects cell functions, generates endothelial dysfunction, and reduces NO synthesis; the reduced bioavailability of NO exerts atherogenic effects [69]. A significant factor that has not been deeply explored is how oxidative stress modulates ion channel oxidation in VSMCs during the development of atherosclerosis. Ion channels represent transcendental elements for the proper function of VSMCs; if their function is compromised, it is important to elucidate how this affects disease development [70]. 

ROS and RNS can affect ion channels directly or indirectly: directly by producing post-translational modifications on the proteins, such as nitrosylation, sulfhydration, or the nitration of specific amino acid residues; or indirectly by altering different signaling pathways. Sulfur atoms in cysteine and methionine confer the sensitivity to redox potentials as well as aromatic rings from histidine, phenylalanine, and tryptophan, and hydroxyl groups in tyrosine residues [14]. The high reactivity of the thiol groups of cysteine contributes to the formation of sulfenic acid, sulfinic acid, or sulfonic acid, depending on oxidant concentration and reaction conditions; methionine forms methionine sulfoxide and methionine sulfone; histidine oxidizes to 2-oxohistidine; and tryptophan oxidizes to 5-hydroxytryptophan and oxindolealanine [71]. Cysteine can suffer other modifications as nitrosylation and glutathionylation [70]. For example, in vascular smooth muscle cells, ROS and NO can oxidize voltage-gated calcium channels Cav1.2b cysteine residues within the alpha subunit and produce conformational changes [14].

### 2.4. Damage in Vascular Smooth Muscle Cells

Vascular smooth muscle cells present differences in the expression of signaling proteins, receptors, and ion channels compared to cardiac and skeletal muscle. Its contractility is fundamentally different because VSMCs do not exert an action potential. They are partially contracted at rest, increasing their contractility in response to neuronal, humoral, or endothelial stimulus acting on membrane receptors. This contractility is relatively slow and sometimes can be sustained and tonic [72]. The maintenance of vascular tone is controlled by VSMC membrane potential. Depolarization activates the L-type high voltage-gated calcium channels (Cav1.2) at the plasma membrane, initiating an increase in Ca^2+^ entry [73]. 

The increase in intracellular Ca^2+^ promotes a contractile response by activating calcium calmodulin-dependent myosin light chain kinase (MLCK) and releasing more Ca^2+^ from intracellular calcium stores [74]. On the other side, K^+^ efflux through Ca^2+^-activated K^+^ channels (KCa) modulates the membrane potential indirectly because they limit the flow of Ca^2+^ ions into cells, causing Cav1.2 inhibition [75]. The vascular smooth muscle cell membrane contains many types of channels. Among calcium channels, we have as the main regulators L-type channels, such as Cav1.2b, which is a different isoform from Cav1.2a in cardiac muscle [14]. They are the major regulators of vascular smooth muscle [Ca^2+^]i and contractility. These channels work at two different levels: depolarization and hyperpolarization. The group in charge of depolarization includes the transient receptor potential family (TRP), TRPC3, TRPC6, and TRPM4 channels, and the group promoting hyperpolarization includes significant conductance calcium-activated potassium channels, TRPV4, and Cav3.2 channels. The entry of calcium into the cell is predominantly mediated by L-type channels (Cav1.2b) and, to some extent, T-type Cav3.1/3.3 channels; they control contraction, and their activity is regulated by changes in the membrane potential [76]. 

Other channels in the plasma membrane include chloride channels. They have several functions, including cell volume regulation, transepithelial transport, ion homeostasis, and the regulation of electrical excitability [77]. In smooth muscle cells, the electrochemical potential for chloride is higher than the resting potential. Then, the opening of chloride channels may produce enough depolarization to cause activation on Cav channels and Ca^2+^ influx, which is important for vascular response to mechanical stress [78].

Membrane ion channels of VSM are classified as follows:

(a) Voltage-gated Ca^2+^ channels (VGCC). These channels regulate contraction and gene expression in VSCM. L-type and T-type Ca channels are representative members of this family. When L-type channels are activated, the membrane is depolarized, and calcium ions enter the cytoplasm; then, potassium channels are activated, and membrane hyperpolarization occurs with the subsequent deactivation of VGCCs [79]. The activation of PKG contributes to vasodilation mediated by NO and inhibits Cav1.2 currents [80]. T-type channels may contribute to myogenic tone at low intravascular pressure when smooth muscle cells are relatively hyperpolarized; however, their specific role needs to be further elucidated [81]. 

(b) Ca^2+^-activated K^+^ channels (KCa). These channels are activated with increased Ca^2+^ intracellular concentration; BKCas are the most abundant in VSMCs. Up to now, only two studies have identified small conductance calcium-activated potassium channels (SKCa) in VSMCs from systemic blood vessels [82], and intermediate conductance calcium-activated potassium channels (IKCa) are expressed only in proliferating VSMCs [83].

(c) Voltage-gated K^+^ channels (Kv). They contribute to the resting tone of small coronary arteries and are a dilator influence in coronary circulation. Kv1.X is the main group of voltage-gated potassium channels in the VSMC of coronary microvessels. Identified family members in the vascular system include Kv1.2 and Kv1.5. The high production of peroxynitrite in hyperglycemia affects these Kv channels and impairs vascular smooth muscle dilation [84]. They regulate the pulmonary circulation and regulate vascular remodeling in pulmonary artery smooth muscle cells [71].

(d) Transient receptor potential channel (TRP). Based on homology sequences, this kind of channel is divided into six members: canonical (TRPC1–7), melanostatin (TRPM1–8), vanilloid (TRPV1–6), ankyrin (TRPA1), polycystin (TRPP1–3), and mucolipin (TRPML1–3). Each family has differences in properties and structure [85]. 

Table 1 shows the most representative channels present in VSMC, how they are affected by oxidative stress, the consequences in membrane potential, and how they participate in atherosclerosis.

Additionally, smooth muscle excitability is modulated indirectly by cytoplasmic calcium concentration released from mitochondria and sarcoplasmic reticulum [86].

### 2.5. Natural Compounds for the Treatment of Atherosclerosis

Many plant derivatives are used as drugs; their advantages include fewer secondary effects and reduction of oxidative stress, LDL cholesterol level, and inflammation [87]. The use of natural compounds in atherosclerosis has focused on prevention or treatment to reduce the levels of blood lipids. Among the different tested substances, it has been observed that the consumption of polyphenol compounds such as flavonoids helps to reduce atherosclerosis development because of its potent antioxidant activity [88]. The Mediterranean diet, which includes the consumption of olive oil and nuts, reduces cardiovascular disease incidence by 30% compared to low-fat diets related to its high phenolic content [89]. The reduction of the atherosclerotic lesion area has been observed with flavonoid treatments in different ex vivo studies in aortas of modified Apo E mice [90,91,92,93,94]. A summary of the various beneficial effects of flavonoids in cardiovascular diseases is shown in Table 2.

## 3. Flavonoids in Atherosclerosis

### 3.1. General Concepts

#### 3.1.1. Classification and Structure

**Figure 2:**
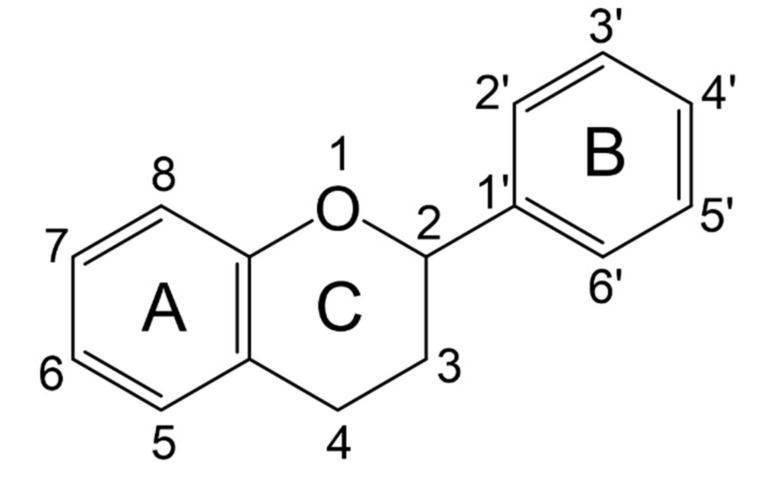
Basic structure of flavonoids.

Flavonoids have a basic structure that consists of two aromatic or phenyl rings, A and B, and one heterocyclic ring C; the last ring is formed with an oxygen atom (Figure 2). Their basic structure contains 15 carbons that can be abbreviated as C6-C3-C6 [12,102], and they may have more than one substituent forming different compounds because the flavonoid’s basic structure may suffer modifications. These modifications include the increase or decrease in the number of hydroxyl groups, flavonoid core, or hydroxyl groups methylation, ortho hydroxyl groups methylation, dimerization, the formation of bisulfates, and hydroxyl groups glycosylation to produce flavonoids O-glycosides or the glycosylation of flavonoid’s cores to produce flavonoids C-glycosides. Most of them belong to the following groups: chalcones, aurones, flavanols, catechins, flavones, flavonols, flavanones, isoflavones, and anthocyanidins. Some characteristics to distinguish them based on their structure, i.e., isoflavones, have the B ring in position 3 of the C ring [103] (Table 3).

#### 3.1.2. Flavonoids Diet Source and Absorption

Anthocyanidins are commonly found in plant pigments, while flavanols are in fruits and tea, flavonols in vegetables and fruits, flavanones in citrus, flavones in vegetables, isoflavones in legumes, chalcones in vegetables and fruits, and aurones in flowering plants. However, their physiological effects depend on their bioavailability, beginning with the absorption process. In general, we consume higher quantities of anthocyanins, flavonols, flavan-3-ols, and flavanones. The natural form of flavonoids in plants is glycosides. We consume them as β-glycosides, except for catechins. Enzymes hydrolyze these compounds in the brush border of small intestine epithelial cells. The released aglycones are lipophilic, and they can cross membranes by passive diffusion into cells without the help of transporters; however, permeability levels depend on size and hydrophobicity. Before they pass into the bloodstream, they are metabolized by enzymes and converted to sulfate, glucuronide, and/or methylated metabolites. The absorption for most of them occurs in the small intestine (Table 3). If not absorbed, they move into distal intestinal portions where interaction with the microbiota and production of other metabolites takes place [104,105]. Aurones have been used for dye and drug development; their predicted absorption is in the intestine demonstrated by in silico pharmacokinetic ADMET parameters [106].

**Table 3:** Groups of flavonoids, general characteristics.

Groups of Flavonoids	Structure	Description	Diet Source and Site of Absorption	Examples	References
Chalcones	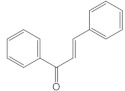	They have ring C open, without the oxygen bridge	Vegetables, fruits, and tea. Small intestine	Isoliquiritigenin, chalconaringenin, phloretin	[104,107,108]
Aurones	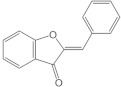	Rings A and C form a benzofuranone	Flowering plants. Intestine	Aureusidin, leptosidin	[106,107]
Anthocyanidins	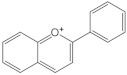	They have at least one -OH group attached in position C3 and C4′, and full conjugated double bonds	Plant pigments of fruits, flowers, leaves, red wine. Small intestine	Cyanidin, malvidin, pelargonidin	[104,107,109]
Flavanols	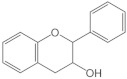	They have a single bond between C2 and C3 and an -OH group at C3. They are also known as flavan-3-ols	Pears, apples, grapes, cocoa, and tea. Oral and small intestine	Catechin, epicatechin, epicatechin derivatives	[104,107]
Flavonols	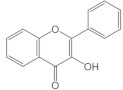	They have the 3-hydroxyflavone backbone (-OH at C3)	Onions, broccoli, tea, spinach, kale, fruits. Proximal small intestine	Quercetin	[104,107,109]
Flavanones	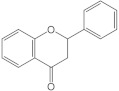	They have a single bond between C2 and C3 and present a carbonyl group at C4	Citrus fruits peels. Small intestine and colon	Eriodictyol, hesperetin, naringenin	[104,109]
Flavones	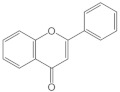	They present a carbonyl group at C4 and do not have an -OH group at C3	Celery, garlic, and chamomile tea. Oral, small intestine, and colon	Apigenin, luteolin	[105,109]
Isoflavonoids or Isoflavones	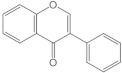	Ring B (Phenyl group) is attached at C3	Soy, and legumes. Colon	Genistein, daidzein	[104,109]

#### 3.1.3. Antioxidant Mechanisms of Flavonoids

The characteristic flavonoid structure gives them antioxidative properties. In some cases, they combat two targets simultaneously; for example, it has been observed that an inhibition of cholesterol-LDL oxidation [110,111] and platelet aggregation can occur with only one compound [112]. In other cases, they inhibit oxidases, i.e., lipoxygenase and cyclooxygenase [113,114], or make a transition metal chelation of iron or copper [115], regulating metal blood levels [116].

**Figure 3:**
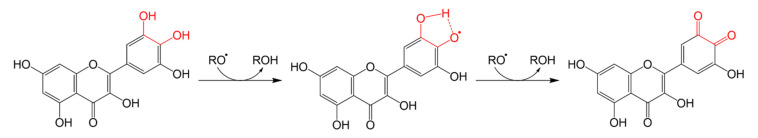
Scavenging of ROS by flavonoids, myricetin neutralizing free radicals, and scavenging of ROS by C3′ and C4′ or C4′ and C5′ di-OHs.

The intake of flavonoids in a healthy diet is higher than other antioxidants such as vitamins C or E and carotenes [117]. Some flavonoids have a great capacity to act on free radicals neutralizing them by electron donation and hydrogen transfer; this is the case of quercetin and myricetin because they have ortho hydroxyl groups in ring B at position C3′ and C4′, or C4′ and C5′ (Figure 3). This characteristic, together with the flavonol structure, gives them a better antioxidant capacity [118].

**Figure 4:**
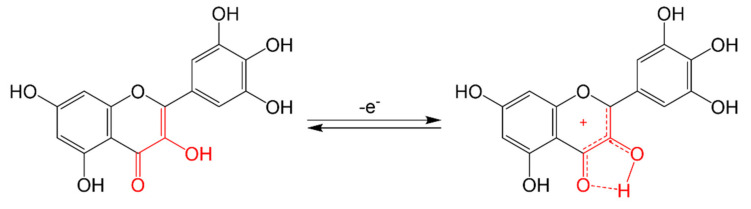
Antioxidation mechanism of C3 and/or C5-OH flavones.

Another antioxidant mechanism is possible for any C3-OH or C5-OH flavone by electron donation where a tautomeric form can behave as an antioxidant in vivo by inhibiting pro-oxidant enzymes (Figure 4) [119].

**Figure 5:**
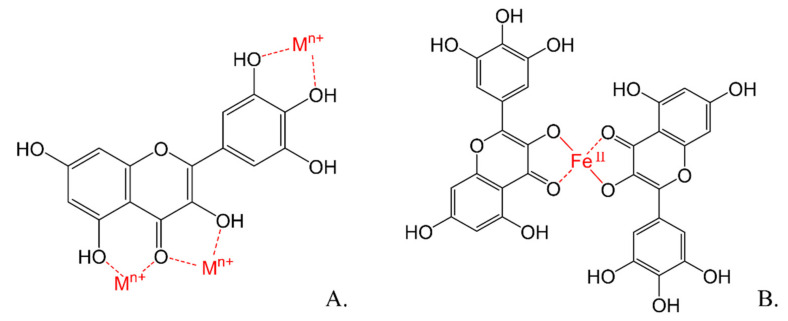
(**A**) Possible sites for trace metals binding. (**B**) Chelation of Fe, forming a chemical complex of myricetin.

Ferric ion chelators prevent the binding of iron to components of the membrane and prevent the precipitation of Fe(OH)_3_; this process avoids hydroxyls radicals or peroxides formation (Figure 5) [120].

**Figure 6:**
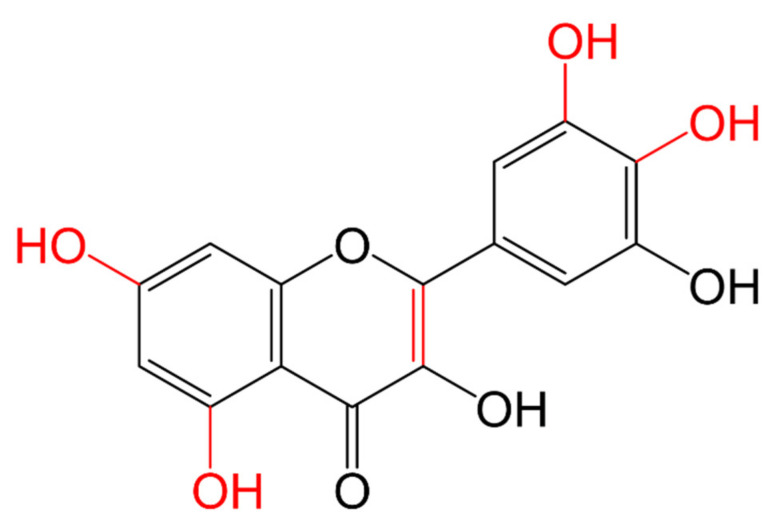
Structural requirements (marked in red) for xanthine oxidases inhibition.

Some requirements have been described for flavonoids to have the ability to inhibit some oxidases, such as the OH group at least at C7 or one additional OH at C5, including a double bond between C2 and C3 in the benzopyrone ring. The catechol group in the B ring could be present to have inhibitory activity on xanthine oxidase (Figure 6). This enzyme catalyzes the oxidation of xanthine and hypoxanthine to uric acid [121,122,123]; this can be used as the base to synthesize inhibitors for this enzyme.

**Figure 7:**
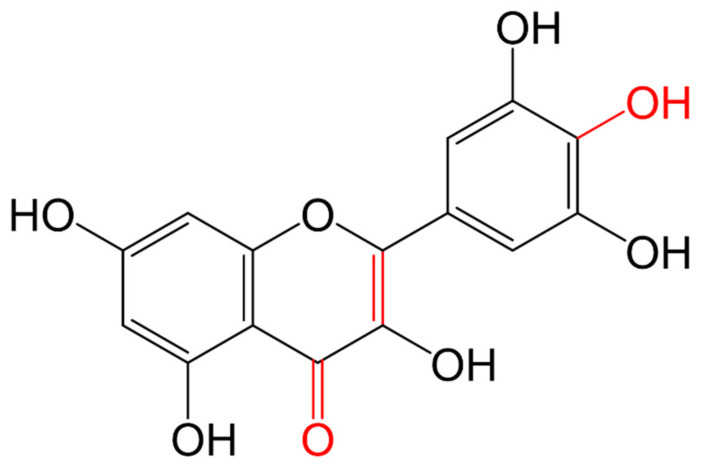
Structural requirements (marked in red) for lipoxygenase inhibition.

Flavonoids can inhibit lipoxygenases if they fulfill structural specifications such as a double bond between C2 and C3, a carbonyl group in C4, and a catechol group in the B ring (OH in C4′ is fundamental, in combination with OH in C3′ or C5′). An excess of OH groups lowers the lipophilic affinity of flavonoids (Figure 7) [124].

**Figure 8:**
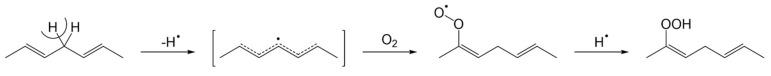
Lipoxygenase reaction.

It is known that aglycones can protect lipids, since the flavonoids without glycosides groups are less water-soluble, more reactive, and they can be closer to lipids than glycosyl-flavonoids. They can participate in a lipoxygenase reaction donating hydrogen with one electron in the last step of the reaction to get a stable lipid that was previously oxidized (Figure 8) [125,126].

### 3.2. Effect of Flavonoids in Atherosclerosis

The consumption of flavonoids in a regular diet has been associated with reducing risk factors in atherosclerosis, which is probably because of their antioxidant and vasoactive properties [127]. The beneficial effects are related to vascular health, including inhibition of LDL oxidation [128], anti-platelet activity [129], reduction of the atherosclerotic lesion [130], lowering blood pressure [131], better endothelial function [132], and improving vascular smooth muscle functions [133]. Effects on VSMC could be related to ion channels activity modulation, since the effect exerts vasodilation in most cases. The effect of apigenin or dioclein on potassium channels reduces their activity and produces vasorelaxation. Other flavonoids produce full vasorelaxation, for example, flavones and flavanones such as acacetin, chrysin, apigenin, hesperetin, pinocembrin, luteolin, 4′-hydroxyflavanone, 5-hydroxyflavone, 5-methoxyflavone, 6-hydroxyflavanone, and 7-hydroxyflavone; partial relaxation is observed with quercetin, quercitrin, hesperidin, and rhoifolin; and some of them do not produce relaxation such as quercetagetin and baicalein [134].

The anti-atherosclerosis effect has been studied mainly in two major groups of flavonoids: flavonols and flavan-3-ols because they are the most abundant compounds in the human diet. They are also structurally similar; both contain a hydroxyl group at C3; however, flavonols contain a carbonyl group at C4 and a double bond between C2 and C3 from the heterocyclic ring, while flavan-3-ols do not. Their effect has been studied in many biological activities with the following findings: LDL oxidation was reduced ex vivo, using quercetin and glabridin [93,94], serum LDL-oxidation in apoE-/- mice was reduced with myricitrin treatment [91], aortic ROS was reduced with kaempferol [92], and plasma fat concentration was reduced with quercetin [135].

Flavonoids diminish oxidative stress by scavenging free radicals and reactive oxygen species [136], downregulating cyclooxygenases and lipoxygenases [137,138,139], upregulating cellular antioxidants [140], and improving anti-inflammatory actions [141]. In the progress of atherosclerosis, flavonoids can avoid thrombus formation and improve lipid and glucose metabolism [142,143,144].

When we consume flavonoids, we metabolize them into glycosides or aglycones. Aglycones are more liposoluble and capable of interacting with cell membranes than glycoside flavonoids [145,146]. This characteristic helps them to be in contact with ion channels.

### 3.3. Effect of Flavonoids in VSMC’s Ion Channels

Ion channels on the plasma membrane of VSMC are affected by flavonoids. The modulation depends on which flavonoid exerts its effect on them. Smooth muscle cell membrane potential is modulated directly by the movement of calcium ions from the extracellular compartment into the cytoplasmic space and indirectly by calcium release from sarcoplasmic reticulum and mitochondria, as we mentioned before [86].

Proper amounts of dietary flavonoids influence the development of cardiovascular diseases by protecting the bioactivity of endothelial nitric oxide. Flavonoids also interfere with the signaling cascades of inflammation. They can prevent the overproduction of NO and its harmful consequences. In healthy tissues, flavonoids can increase endothelial nitric oxide synthase (eNOs) activity, which is necessary to produce vasodilation. In oxidative stress and inflammatory conditions, flavonoids inhibit the NFkB pathway to prevent inflammation. Flavonoids reduce peroxynitrite and superoxide levels and prevent the overexpression of ROS-generating enzymes [147].

Fusi et al. (2017) studied by docking analysis the interaction between flavonoids and the Cav1.2 channel α1c subunit. They analyzed two groups of flavonoids; the first group inhibited calcium currents: scutellarein, morin, 5-hydroxyflavone, trihydroxyflavone, (±)-naringenin, daidzein, genistein, chrysin, resokaempferol, galangin, and baicalein, and the second group stimulated calcium currents: myricetin, quercetin, isorhamnetin, luteolin, apigenin, kaempferol, and tamarixetin. This study showed differences between flavonoid interactions; epigallocatechin gallate affects Cav1.2 currents in an endothelium-independent manner, while epicatechin gallate does not affect them. Hesperetin and cardamonin block Cav1.2 channels and increase Kv currents, producing vasorelaxation. At the same time, kaempferol 3-O-(6′-trans-p-coumaroyl)-β-d-glucopyranoside (tiliroside) causes a partial inhibition of Cav1.2 channels in vascular smooth muscle [148].

Other possible mechanisms that influence atherosclerosis include the effect of flavonoids on ion channels for blood pressure regulation. Marunaka (2017) reports a quercetin activity outside vascular tissue that stimulates Na^+^–K^+^–2Cl^−^ cotransporter 1 (NKCC1), regulating the cytosolic Cl^−^ concentration in lung endothelial cells. The elevated chloride concentration downregulates the expression of epithelial Na^+^ channels, controlling blood volume by Na^+^ reabsorption with a consequent decrease in blood pressure [149].

Recently, Fusi et al. (2020) studied the beneficial effects of flavonoids on the cardiovascular system, emphasizing the study of potassium channels by docking analysis. They describe flavonoid–channel interactions at the molecular level and relate them with experimental evidence. They observed that the main vasodilator effects are associated with the opening of K^+^ channels. In some experiments, the effect is dose-dependent; for example, baicalin at daily doses of 50 to 200 mg/kg body weight lowers blood pressure in an experiment with hypertensive rats due to ATP-dependent K^+^ (K_ATP_) activation [150].

## 4. Effects of Flavonoids on Atherosclerosis through Modulation of Ion Channels in VSMC Activity

Flavonoids can exert effects on different ion channels in VSMC and produce changes in the progression of atherosclerosis. Effects can modulate ion channel activity and make changes in ion currents and vascular tone. Several flavonoids inhibit calcium currents, producing vasorelaxation; this is the case of genistein, phloretin, and biochanin-A, which act through an endothelium-independent mechanism; this mechanism does not involve ATP-sensitive potassium channels but may involve other channels [151]. Scutellarin relaxes rat aortic rings in a dose-dependent form by inhibiting calcium currents; this process is independent of voltage-dependent calcium channels, demonstrating the participation of other calcium channels for calcium influx mediation during contraction. The candidates for this action include non-selective cation channels, receptor-operated calcium channels (ROCCs), and store-operated calcium channels (SOCCs), among others. As a result of this effect, scutellarin is used to treat ischemic diseases or hypertension related to atherosclerosis [152]. Other biological activities related to relaxant flavonoid actions are anti-platelet aggregation and inhibition of smooth muscle cell proliferation [153]. Daidzein, genistein, apigenin, and trans-resveratrol inhibit SOCCs and impede platelet aggregation and thrombus formation, with an effect that is related to second messengers [154].

Epigallocatechin from green tea can act at two levels: first, increasing calcium influx to generate endothelium-independent vasoconstriction, and second, by inhibiting voltage-gated calcium channels to induce vasodilation. Long treatments of 200 mg/kg/day of epigallocatechin significantly reduce systolic blood pressure in spontaneously hypertensive rats; in normotensive rats, effects were shown at a dose of 25–100 mg/kg/day [155,156]. (−)-Epigallocatechin-3-gallate and (−)-epicatechin-3-gallate reduce the activity of K_ATP_ channels at low concentrations, but higher concentrations completely inhibit the channel [157]. Quercetin is a flavonoid that activates L-type Ca^2+^ channels in VSMCs; however, quercetin-induced vasorelaxant mechanisms are more relevant than the increase in Ca^2+^ influx. On the other hand, rutin, the glycoside form of quercetin, acts only during endothelium-dependent relaxation due to its lower liposolubility [158]. Quercetin decreases the cell surface expression of vascular cell adhesion molecules and reduces lipid peroxidation [109]. The significant quercetin effects are observed in resistance arteries compared to conductive arteries [107].

Activation of calcium-activated potassium channels is a key mechanism in flavonoid-induced vasorelaxation. Kaempferol activates BKCa channels of endothelial cells, resulting in membrane hyperpolarization, and this mechanism contributes to vasodilation [159], while puerarin activates BKCa channels on smooth muscle cells, resulting in vasodilation [160]. Dioclein generates hypotension in normal rats, which is caused by the opening of the KCa channels [161]. Saponara et al. (2006) demonstrated that naringenin activates BKCa channels and dilates aortic rings [162]. The same results were obtained with quercetin, puerarin, epigallocatechin, and proanthocyanidins through ion channel activation, hyperpolarization, and vasorelaxation [162,163,164]. The contribution of BKCa agonists in atherosclerosis is to lower blood pressure and improve other cardiovascular symptoms [160].

Genistein inhibits Kv current with the slow recovery of voltage-gated potassium channels [165]. The activation of potassium channels shows vasodilatory effects. Tilianin produces vasorelaxation that may be produced due to an opening of these potassium channels [166]. Kolaviron, amentoflavone, pinocembrin, luteolin, and cardamonin act via two effects: firstly, by reducing calcium currents and, secondly, by increasing potassium currents, both increasing vasodilation [167,168,169,170,171].

Calderone et al. (2004) investigated the endothelium-independent vasorelaxant effect of flavonoids mediated by potassium channels. Their results showed that two flavonoids were almost entirely ineffective: baicalein and quercetagetin. Quercetin, quercitrin, rhoifolin, and hesperidin had partial vasorelaxant effects, while the rest showed full vasorelaxant effects, such as acacetin, apigenin, chrysin, hesperetin, luteolin, pinocembrin, 4′-hydroxyflavanone, 5-hydroxyflavone, 5-methoxyflavone, 6-hydroxyflavanone, and 7-hydroxyflavone, all of them belonging to flavanones and flavones groups. The study concluded a relationship between the flavonoid structure and large-conductance, calcium-activated potassium channels. It seems that the presence of the C5-OH group is necessary for the interaction and also for the involvement of ATP-sensitive potassium channels [134].

On the other hand, acacetin prevents atrial fibrillation, inhibits ultrarapid delayed rectifier potassium currents, and blocks the acetylcholine-activated potassium current, achieving the prolongation of the action potential and the effective refractory period, preventing atrial fibrillation [172]. Studies have shown that isoliquiritigenin inhibits atherosclerosis by blocking TRPC5 channel expression in VSMCs. This store-operated channel activates the transcription of early response genes to proliferate and migrate [108].

**Figure 9:**
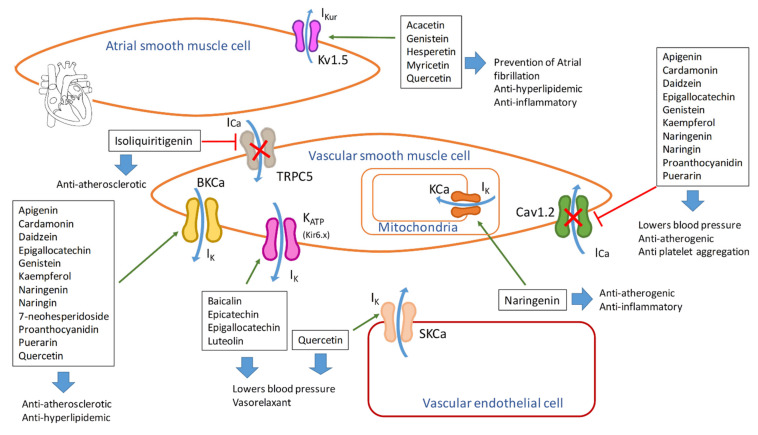
Actions of flavonoids on ion channels of cells from the cardiovascular system.

Table 4 describes the effects of flavonoids on ion channels and their impact on atherosclerosis progression; Figure 9 depicts the localization of ion channels summarizing flavonoids’ effects.

Endothelial, atrium smooth muscle, and vascular smooth muscle cells are presented. Channels are inhibited (red line) or stimulated (green arrow) by flavonoids, resulting in different effects during atherosclerosis progression. I_Kur_: ultrarapid delayed rectifier K^+^ currents; IK: potassium currents; ICa: calcium currents; Kv1.5: voltage-dependent potassium channel; BKCa: large-conductance calcium-activated potassium channel; K_ATP_: ATP activated potassium channel; Cav1.2: voltage-dependent calcium-channel; SKCa: small conductance potassium channel; KCa: calcium-activated potassium channel; TRPC5: transient receptor potential canonical 5 channel.

## 5. Future Perspectives in the Treatment

The harmful effects of oxidants have been acknowledged for decades, and many pathogenic mechanisms have been identified in numerous diseases. The case of atherosclerosis is a typical example since disease progression would not take place without the oxidation of lipids, as has been extensively reviewed here. However, under oxidative stress conditions, lipids are not the only affected molecules. The role of other altered molecular structures needs to be considered for proper physiopathology comprehension and future drug design. With this review, we tried to emphasize the role of voltage-gated ion channels in VSMCs. Membrane potential regulation is transcendental for muscle function and depends on the proper function of each ionic conductance. There are still many unanswered questions about the specific role of the oxidized channels during the onset and development of atherosclerosis. Unraveling specific pathogenic mechanisms of each channel type will open new therapeutic targets that could prevent cardiovascular complications. Here, we have shown the major ion channels affected by oxidation; further efforts to describe how and when their misfunction affects disease development are needed.

On the other hand, the beneficial effects of foods widen our options toward finding new natural compounds that can be used at different stages of atherosclerosis. Even though antioxidative, antithrombotic, anti-inflammatory, and vasorelaxant mechanisms of flavonoids are known, the scope of their benefits needs to be enlarged to new molecular targets that are not usually considered. As shown in Table 4, the effects of flavonoids on ion channels have been extensively described; however, the connection between their functional restoration and disease improvement needs to be approached in detail.

The antioxidant mechanisms of flavonoids are considered part of medicinal chemistry; it is necessary to deepen their structural and functional relationship and the role of pharmacokinetics and pharmacodynamics for their effect [173]. Nanotechnology may play a key role shortly to improve the bioavailability of the compounds. Future work with the aid of network pharmacology approaches will be needed to find significant targets in the treatment of atherosclerosis. In the case of quercetin, one of the most studied flavonoids, a recent network pharmacology study identified 47 cardiovascular disease-related targets and 12 pathways of the Kyoto Encyclopedia of Genes and Genomes, which may even display synergistic therapeutic effects. Studies such as docking analysis will unravel the precise mechanisms by which flavonoids interact with specific lipids and protein targets [174]. Our work demonstrates how nutritional and traditional medicine may be combined with sophisticated bioinformatical approaches to show specific molecular targets of natural compounds with high precision to support drug development.

## 6. Conclusions

In conclusion, flavonoids have direct or indirect effects over ion channels and vascular smooth muscle function; they are vasodilator compounds, antioxidants, reduce peroxidative reactions, inhibit platelet aggregation, and decrease thrombotic tendency.

Among these activities, they have the antioxidant capacity to protect LDL, reducing reactive oxygen species and oxidizing enzymes, their activity of trapping metal ions, reinforcing the endogenous antioxidant capacity. Combining those actions, working on different targets, including ion channels, affects the development of atherosclerosis in a significant way, improving vascular smooth muscle function.

## Figures and Tables

**Table 1:** Oxidation changes in ion channels and their effect on atherosclerosis.

Ion Channel	Molecular Target of ROS	Physiological Effect	Membrane Potential Effect	Effect on the Disease	Expression
L-type Cav 18–25 pS (Ca_v_ 1.2b)	Cysteine oxidation within alpha subunit, C1789, C1790, C1810 on alpha1C	↓ Ca^2+^ currents	Lack of depolarizing stimuli. Reduced sensitivity toward physiological stimulation	Reduction in dihydropyridine binding sites. Less vasocontraction	Cav1.2b expression is not affected. Smooth muscle contractility alterations
T-type Cav 8 pS (Ca_v_ 3.1–3.3)	Extracellular cysteine oxidation	↓ Ca^2+^ currents	Non-significant effects on membrane potential	Diminish Vasocontraction	ND
BKca 200–300 pS	Slo1 Cysteines oxidation residues: C14, C141, C430, C615, C911 and methionine residues: M536, M712, M739	Decreased potassium outward currents ↓ [Ca^2+^]i	Abnormally depolarized resting membrane potential	Decreased Slo channel activity. Inactivation. Alters membrane function, losing homeostasis, and leading to disease. Less vasocontraction	Downregulated
IKCa 20–90 pS	ND	Decreased potassium outward currents ↓ [Ca^2+^]i	Abnormally depolarized resting membrane potential	SMCs proliferation and migration	Upregulated
SKCa 5–20 pS	ND	Decreased potassium outward currents ↓ [Ca^2+^]i	Abnormally depolarized resting membrane potential	SMCs proliferation and migration	Upregulated
Kv1.X	ND	Decreased currents ↓ [Ca^2+^]i	Abnormally depolarized resting membrane potential	Alters membrane function, losing homeostasis and leading to disease. Less vasocontraction	ND
TRPM4	Oxidation of a Cysteine terminally, C1093	↑ Ca^2+^, Na^+^ influx desensitization	Membrane depolarization	↑ Necrosis and cell death Opening of Cav1.2 channels resulting in SMC contraction	Cell-specific upregulated
TRPC3	ND	↑ Na^+^ influx	Membrane depolarization	ND	Cell-specific upregulated
TRPC6	ND	↑ Na^+^, Ca^2+^ influx	Membrane depolarization	Opening of Cav1.2 channels resulting in SMC contraction	Cell-specific upregulated

**Table 2:** Plants as natural sources of flavonoids with therapeutic effects for cardiovascular diseases and atherosclerosis treatment.

Plant	Flavonoids	Therapeutic Effect	References
*Polygonum minus* (*Persicaria minor*) (pygmy smartweed, small water pepper, or swamp willow weed)	Myricetin, quercetin, methyl-flavonol	Antioxidant, anti-inflammatory	[95,96]
***Ajuga Iva*** **(L.)** **(búgula almizclada)**	Naringenin, apigenin-7-O-neohesperidoside	Antioxidant, anti-inflammatory, anti-hypercholesterolemia	[97]
*Abelmoschus esculentus* (ladies’ fingers or ochro)	Quercetin	Anti-inflammatory, antioxidant, hypolipidemic	[95]
*Astragalus membranaceus* (Mongolian milkvetch)	Total flavones	Inhibition of foam cell formation	[97]
*Engelhardia roxburghiana* (yellow basket-willow or roxburgh engelhartia)	Total flavonoids, naringenin, kaempferol, quercetin, isoengeletin, engeletin, astilbin, quercitrin	Decreased the serum lipids, downregulated NF-κB signaling	[98]
*Scutellaria strigillosa* Hemsley(namikiso (Japanese meaning: coming wave weed))	Wogonin, wogonoside, baicalein, baicalin	Anti-proliferative and anti-migratory vascular smooth muscle cells	[99]
***Garcinia madruno*** **(madruno, charichuela, or madrono)**	Pure biflavonoid aglycones morelloflavone, volkensiflavone	Protect low-density lipoprotein particle from both lipid and protein oxidation	[100]
*Pandanus tectorius* (pandan laut, tahitian screwpine, or pandanus)	Tangeretin	Anti-hypercholesterolemia	[101]

**Table 4:** Flavonoids and their advantages in atherosclerosis.

Flavonoid	Ion Channel Target	Physiological Effect	Effect on Membrane Potential	Advantages in Atherosclerosis
Acacetin Apigenin trimethylether Genistein Hesperetin Myricetin Quercetin	Kv1.5	Channel inhibitor	Decreased currents Inhibits ultrarapid delayed rectifier K^+^ currents (Ikur)	Prevention of atrial fibrillation Anti-hyperlipidemic, anti-inflammatory
Apigenin Cardamonin Daidzein Epigallocatechin Genistein Kaempferol Naringenin Naringin Proanthocyanidin Puerarin Quercetin	KCa1.1 (BKCa)	Channel activation Vasorelaxant	Increased currents, Hyperpolarization	Anti-atherosclerotic Anti-hyperlipidemic
Quercetin	SKCa	Channel activation Vasodilator effect	Increased currents, endothelial hyperpolarization, direct electrical coupling with VSMC hyperpolarization	Lowers blood pressure
Baicalin Epicatechin Epigallocatechin Luteolin	K_ATP_	Channel activation Vasorelaxant	Increased currents Hyperpolarization	Lowers blood pressure
Epicatechin gallate Epigallocatechin gallate Genistein	Kir6.1	Channel inhibitor	Decreased currents	Anti-inflammatory
Naringenin	Mitochondrial KCa	Vasodilator effect	Hyperpolarization	Anti-atherogenic, anti-inflammatory
Cardamonin Chrysin Daidzein Epigallocatechin gallate Galangin Genistein Hesperetin Kaempferol Morin Quercetin Scutellarein Tiliroside	Cav1.2	Channel inhibitor Vasorelaxant	Decreased Ca currents	Lowers blood pressure Anti-atherogenic Anti-platelet aggregation
Isoliquiritigenin	TRPC5	Channel inhibitor	Decreased Ca currents	Anti-atherosclerotic
Quercetin	NKCC1 (lung endothelial cells)	Channel activator	Increased Cl^−^ currents	Lowers blood pressure by diminishing the expression of αENaC in renal cells

BKCa: big conductance calcium-activated potassium channel (KCa1.1); K_ATP_: ATP-sensitive potassium channel; KCa: calcium-activated potassium channel; Kv: voltage-gated potassium channel; Kir: inward rectifier potassium channel; Cav: voltage-gated calcium channel; NKCC1: cation–chloride cotransporter (Na^+^–K^+^–2Cl^−^– cotransporter 1); TRPC5: transient receptor potential canonical 5 channel.
